# NAC Attenuates LPS-Induced Toxicity in Aspirin-Sensitized Mouse Macrophages via Suppression of Oxidative Stress and Mitochondrial Dysfunction

**DOI:** 10.1371/journal.pone.0103379

**Published:** 2014-07-30

**Authors:** Haider Raza, Annie John, Jasmin Shafarin

**Affiliations:** Department of Biochemistry, College of Medicine and Health Sciences, United Arab Emirates University, Al Ain, United Arab Emirates; Wayne State University School of Medicine, United States of America

## Abstract

Bacterial endotoxin lipopolysaccharide (LPS) induces the production of inflammatory cytokines and reactive oxygen species (ROS) under in vivo and in vitro conditions. Acetylsalicylic acid (ASA, aspirin) is a commonly used anti-inflammatory drug. Our aim was to study the effects of N-acetyl cysteine (NAC), an antioxidant precursor of GSH synthesis, on aspirin-sensitized macrophages treated with LPS. We investigated the effects of LPS alone and in conjunction with a sub-toxic concentration of ASA, on metabolic and oxidative stress, apoptosis, and mitochondrial function using J774.2 mouse macrophage cell line. Protection from LPS-induced toxicity by NAC was also studied. LPS alone markedly induced ROS production and oxidative stress in macrophage cells. When ASA was added to LPS-treated macrophages, the increase in oxidative stress was significantly higher than that with LPS alone. Similarly, alteration in glutathione-dependent redox metabolism was also observed in macrophages after treatment with LPS and ASA. The combination of LPS and ASA selectively altered the CYP 3A4, CYP 2E1 and CYP 1A1 catalytic activities. Mitochondrial respiratory complexes and ATP production were also inhibited by LPS-ASA treatment. Furthermore a higher apoptotic cell death was also observed in LPS-ASA treated macrophages. NAC pre-treatment showed protection against oxidative stress induced apoptosis and mitochondrial dysfunction. These effects are presumed, at least in part, to be associated with alterations in NF-κB/Nrf-2 mediated cell signaling. These results suggest that macrophages are more sensitive to LPS when challenged with ASA and that NAC pre-treatment protects the macrophages from these deleterious effects.

## Introduction

Resident and circulating macrophages present, throughout the human body, play important roles in the determination of inflammation and stress- related abnormalities [1], [2]. Alterations in tissue microenvironment by macrophages and their continuous adjustment have been implicated in the etiology and pathophysiology of numerous diseases. Both cytoprotection and augmentation of cytotoxicity have been reported by macrophages. Oxidative stress and inflammation act as cooperative and synergistic partners in the pathophysiology of numerous diseases such as cancer, diabetes, obesity, cardiovascular and neurological disorders [3], [4]. Lipopolysaccharides (LPS) are a component of the outer wall of gram-negative bacterial endotoxin which penetrates cells and induces inflammatory and other toxic responses [5], [6]. LPS absorption in macrophages and other cells is increased under pathophysiological conditions [7]. LPS induces cellular toxicity by involving inflammatory cells and chemical mediators such as reactive oxygen species (ROS), reactive nitrogen species (RNS), pro-inflammatory cytokines and prostaglandins (PGs) [8], [9]. Acetylsalicylic acid (ASA, aspirin), a specific inhibitor of cyclooxygenase (COX) enzyme and a commonly used anti-inflammatory drug, has multiple pharmacological effects which may not be associated with its COX inhibitory activity [10], [11].

Our previous studies on murine macrophage J774.2 cells and human hepatoma HepG2 cells, treated with either acetaminophen or aspirin, have indicated increased oxidative stress and mitochondrial dysfunction accompanied by increased apoptosis [11]–[13]. Although aspirin is considered to be a specific inhibitor of PGs synthesis, its effect on COX is still controversial in different cell lines and its broad pharmacological effects may be associated through the regulation of redox metabolism, cell signaling and mitochondrial functions [14]–[15]. We, therefore, have studied the effects of LPS stimulation on murine macrophage J774.2 in conjunction with ASA. Results from these studies suggest that ASA sensitizes macrophages towards enhanced LPS-induced toxicity by enhancing cellular oxidative stress and mitochondrial dysfunction. These effects are also associated with altered glutathione (GSH)-redox homeostasis. NAC pre- treatment, however, protected the macrophages from LPS-induced toxicity.

## Materials and Methods

### Materials

Aspirin, LPS, NAC, malondialdehyde, NADPH, 2-thiobarbituric acid, reduced glutathione (GSH), 1-chloro 2,4-dinitrobenzene (CDNB), cumene hydroperoxide, glutathione reductase, 5,5′-dithio bis-2-nitrobenzoic acid (DTNB), cytochrome c, coenzyme Q2, antimycin A, dodecyl maltoside, N-nitrosodimethylamine (NDMA), erythromycin, 7-ethoxyresorufin, resorufin and ATP bioluminescent somatic cell assay kits were purchased from Sigma (St Louis, MO, USA). 2,7-Dichlorofluorescein diacetate (DCFDA) was purchased from Molecular Probes, Inc. (Eugene, OR, USA). Kits for mitochondrial membrane potential assays were procured from R & D Systems, MN, USA. Apoptosis detection kits for flow cytometry and IL6 and TNF-α measurement kits were purchased from BD Pharmingen (BD Biosciences, San Jose, USA). Murine macrophage J774.2 cells, which are circulatory monocyte macrophages from tumor bearing mice, were purchased from European Collection of cell cultures (Health Protection Agency Culture Collections, Salisbury, UK). Polyclonal antibodies against beta-actin, HO-1, TNF-α, Nrf-2, IκB-α, cytochrome c, and Bcl-2 and, NF-κBp65 were purchased from Santa Cruz Biotechnology, Inc (Santa Cruz, CA, USA). Reagents for electrophoresis and Western blot analyses were purchased from Bio-Rad Laboratories (Richmond, CA, USA).

### Cell culture, treatment and cellular fractionation

J774.2 cells were grown in poly-L-lysine coated 75 cm^2^ flasks (∼2.0–2.5×10^6^ cells/ml) in DMEM supplemented with 2 mM glutamine, 10% heat-inactivated fetal bovine serum in the presence of 5% CO_2_-95% air at 37°C as described before [12]. Cells were cultured to 80% confluence and treated with 1 µg/ml E.coli LPS for 24 h. In some cases, the cells were treated with 5 mM ASA for 24 h with LPS, with or without. NAC (10 mM) treated for 2 h prior to LPS treatment. Cells were harvested after the treatments and cellular fractions, mitochondria and post mitochondrial supernatant (PMS) were used for further analyses as described before [11]–[13].

### Measurement of reactive oxygen species (ROS)

J774.2 macrophages cells (1–5×10^5^ cells/well) were cultured in 6-well plates for 24 h prior to LPS treatment. In some experiments, cells were also treated with 5 mM ASA and/or 10 mM NAC (as described above). The intracellular production of ROS was measured by FACS analysis of ASA/LPS-treated and -untreated J774.2 cells using the cell permeable probe, DCFDA, which preferentially measures peroxides as described before [11]–[13]. Reactive oxygen species were also measured microscopically (Olympus fluorescence microscope) using DCFDA-probe. Cells (1–5×10^5^ cells/ml) were grown on cover slips and incubated with 5 µM DCFDA for 30 min at 37°C. Cells were washed twice with phosphate buffered saline (PBS) and fluorescence was immediately analyzed microscopically.

### Measurement of apoptosis

The annexin V externalization assay for apoptosis was performed using flow cytometry after treatment of J774.2 cells with LPS or ASA alone or in combination as described in the vendor's protocol (BD Pharmingen, BD Biosciences, San Jose, USA). Briefly, LPS/ASA-treated and untreated cells from 60%–70% confluent plates were trypsinized, washed in PBS and resuspended (1×10^6^ cells/ml) in binding buffer (10 mM HEPES, pH 7.4, 140 mM NaCl, 2.5 mM CaCl_2_). A fraction (100 µl/1×10^5^ cells) of the cell suspension was incubated with 5 µl annexin V conjugated to FITC and 5 µl propidium iodide (PI) for 15 min at 25°C in the dark. 400 µl of binding buffer was added to the suspension and apoptosis was measured immediately using a Becton Dickinson FACS CantoII analyzer as described before [11]–[13].The apoptotic cells were estimated as the percentage of cells that stained positive for Annexin V-FITC while remaining impermeable to PI (AV+/PI−). This method also distinguished viable cells (AV−/PI−) and cells undergoing necrosis (AV+/PI+).

### Measurement of glutathione (GSH) metabolism

J774.2 macrophage cells were treated with LPS/ASA with or without NAC as described above. GSH concentration was measured by enzymatic recycling method using NADPH and GSH-reductase as described [13].Glutathione S-transferase (GST) activity using CDNB and glutathione peroxidase (GSH-Px) activity using cumene hydroperoxide as substrates were measured by standard protocols as described before [11]–[13].

### Measurement of CYP 450 activities

CYP 3A4, CYP 1A1 and CYP 2E1 activities in control and LPS/ASA-treated macrophage cells were measured using standard substrates, erythromycin, 7-ethoxyresorufin and N-nitrosodimethylamine, respectively by standard methods as described previously [16]–[17].

### Measurement of cytokines

IL6 and TNF-∝ were measured in cells treated with LPS/ASA in the presence or absence of NAC using ELISA kits from BD Pharmingen (BD Biosciences, San Jose, USA) as per the vendor's protocol and the plates were read at 450 nm using the Gen 5 ELx 800 plate reader (Wincoski,VT,USA)

### Measurement of mitochondrial membrane potential (MMP)

Macrophage cells (2–6 ×10^6^ cells/well) were treated with LPS/ASA with or without NAC as described above. The mitochondrial membrane potential was measured according to the vendor's protocol (DePsipher, R &D Systems Inc.) by flow cytometry using a fluorescent cationic dye as described before [13]. DePsipher has the property of aggregating upon membrane polarization forming an orange-red fluorescent (absorption/emission 585/590 nm) compound. If the membrane potential is reduced, the dye cannot access the transmembrane space and remains in its green fluorescent (510/527 nm) monomeric form.

### Measurement of ATP level

Macrophage cells were treated with LPS/ ASA with or without NAC as described above. The ATP content in the cell lysate was determined using ATP Bioluminescent cell assay kit according to the manufacturer's suggestion (Sigma, St Louis, MO) and samples were read using the TD-20/20 Luminometer (Turner Designs, Sunnyvale, CA).

### Measurement of mitochondrial respiratory complexes

Freshly isolated mitochondria (5 µg protein) from LPS/ASA-treated macrophages with or without NAC were suspended in 1.0 ml of 20 mM KPi buffer, pH 7.4, in the presence of the detergent, lauryl maltoside (0.2%). NADH ubiquinone oxidoreductase (Complex I) and cytochrome c oxidase (Complex IV) were measured using the substrates coenzyme Q2 and reduced cytochrome c, respectively by the methods of Birch-Machin and Turnbull [18] as described before [13].

### SDS-PAGE and Western blot analysis

Protein (50–100 µg) from the different sub cellular fractions of control and treated J774.2 cells were separated on 12% SDS-PAGE [19] (Laemmli, 1970) and electrophoretically transferred onto nitrocellulose membrane by Western blotting [20] (Towbin et al., 1979). Transferred proteins were checked by reversible Ponceau S staining for equal loading and then probed with primary antibodies against HO-1, TNF-∝ NrF-2, IκB-∝, NF-κB, Bcl-2 and cytochrome c. Immunoreactive bands were visualized using the appropriate conjugated secondary antibodies. Equal loading of protein was confirmed using beta-actin as the loading control. After development of the blots, the bands were visualized and further densitometric analysis performed using the Typhoon FLA 9500 system (GE Healthcare, Uppsala, Sweden) and expressed as relative intensity (R.I) compared to the untreated control.

### Statistical analysis

Values shown are expressed as mean + SEM of 3 individual experiments. Statistical significance of the data was assessed using SPSS software (version 21) by analysis of variance followed by Dunnett's post-hoc analysis. P values ≤0.05 were considered statistically significant.

## Results

### Effect of NAC on LPS/ASA-induced ROS production

**Figure 1:**
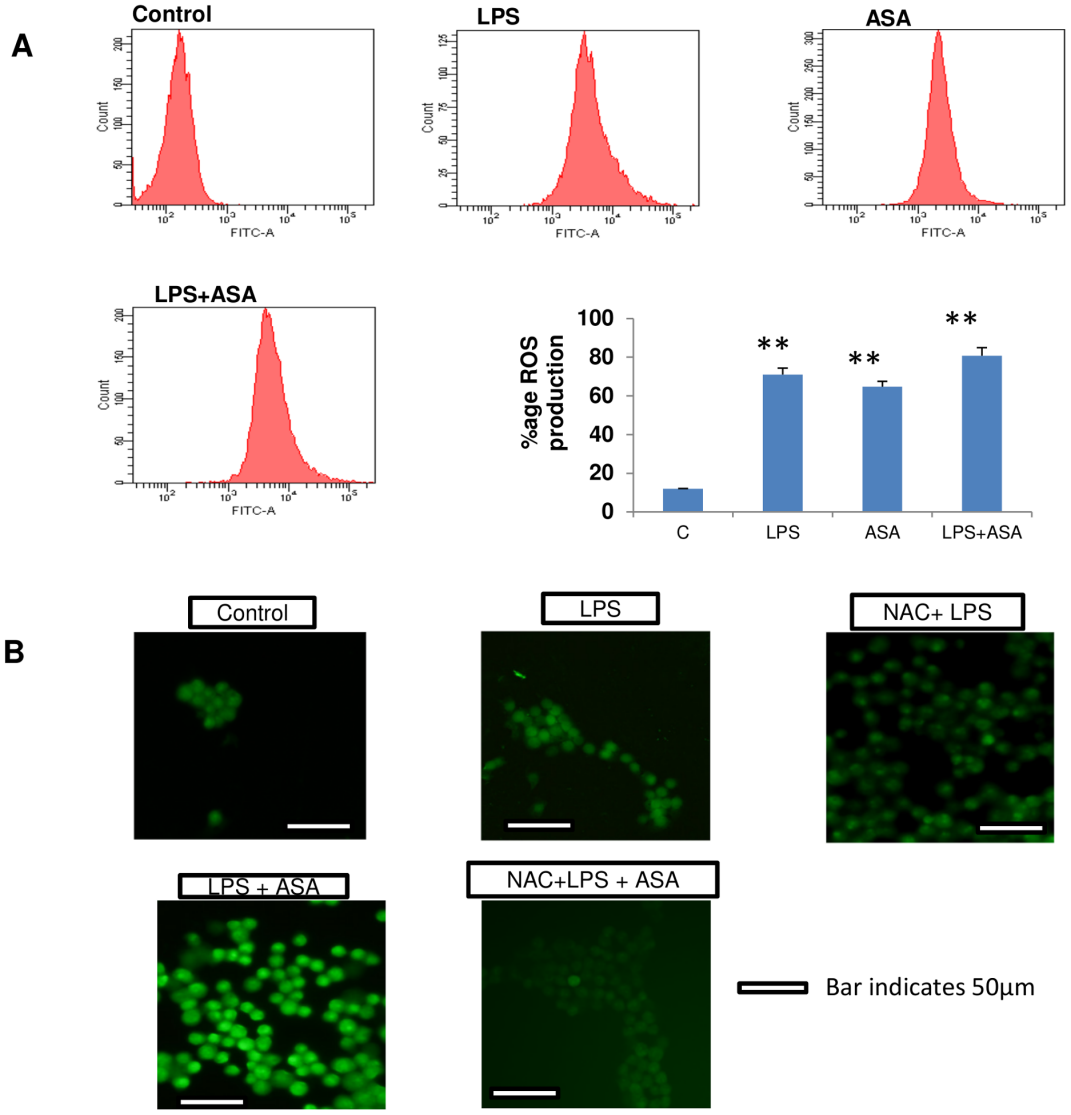
LPS- and ASA-induced ROS production. J774.2 macrophage cells were cultured to 80% confluence and treated with LPS and ASA alone or in combination as described in Materials and Methods. ROS production was measured in cell lysate using DCFDA and fluorescence was measured by flow cytometry (1A) using FACSDiva software as described before [13]. Results are expressed as a typical representation of three determinations. Percent change in ROS production from a typical histogram is also presented. Results are expressed as mean +/− SEM of at least three experiments. Asterisks indicate significant difference (** p ≤0.01) from control (C). In some cases, cells were washed twice with PBS and DCFDA-induced ROS fluorescence was immediately analyzed microscopically using an Olympus fluorescence microscope (1B). Typical results from control and LPS alone or in combination with ASA with or without NAC treatment from three experiments are shown.

Figure1A shows the differential effects of LPS and ASA alone or in combination on ROS production in macrophages. A 6-fold increase in ROS production was observed with LPS and ASA treatment. The combination of LPS and ASA also resulted in about a 6-7-fold increase in ROS production in these cells. Immunofluorescent microscopic studies also showed an increased DCFDA-ROS staining in LPS/ASA-treated macrophages. NAC treatment prior to LPS alone or LPS and ASA treatments resulted in marked reduction in ROS production (Figure 1B).

### Effect of NAC on LPS and ASA-induced apoptosis

**Figure 2:**
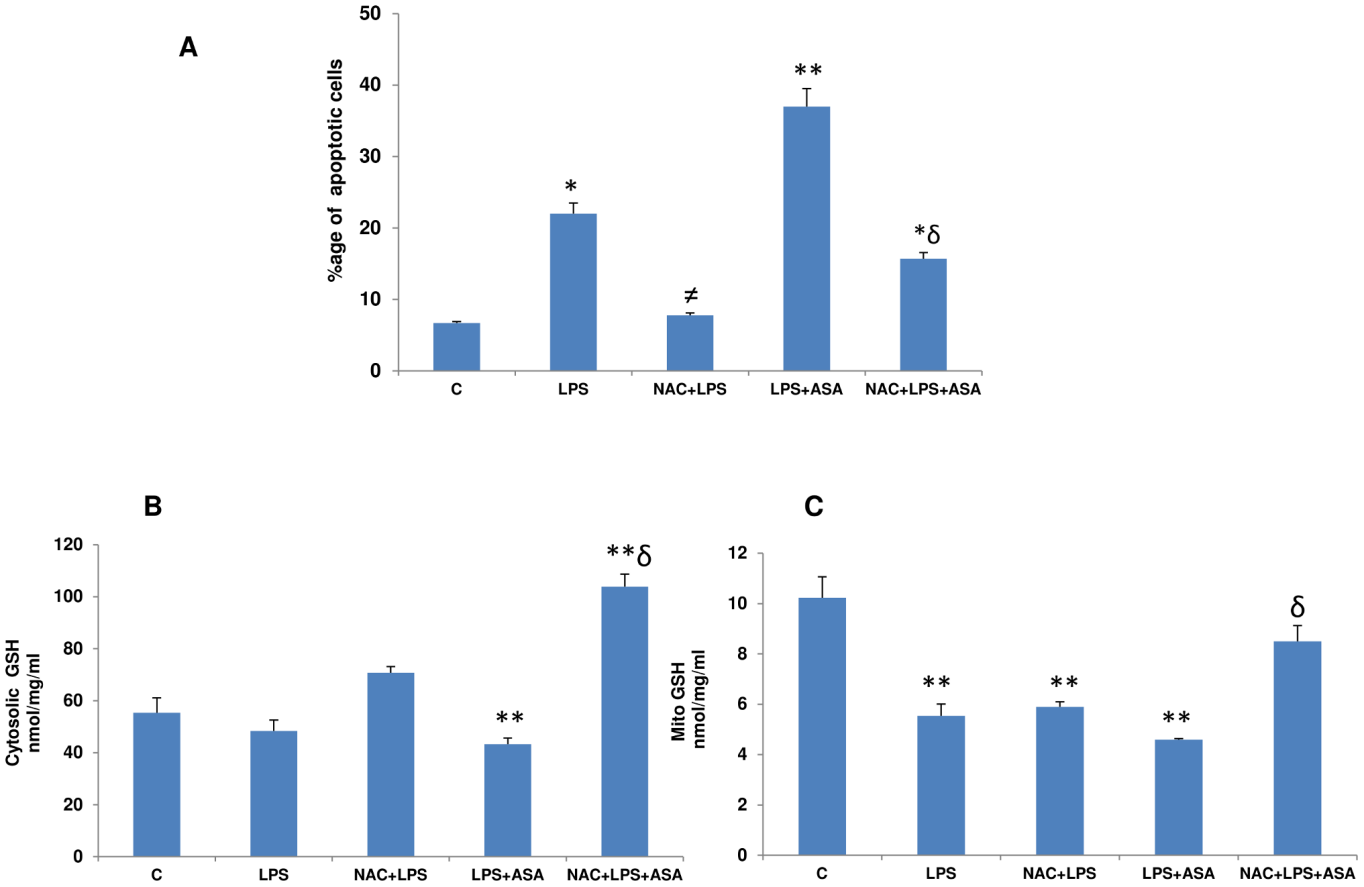
A–C: LPS- and ASA-induced apoptosis and alteration in GSH. Macrophage J774.2 cells were cultured to 80% confluence and treated with LPS alone or in combination with ASA in the presence or absence of NAC as described in Materials and Methods. Apoptosis was measured by flow cytometry as described in Materials and Methods. Bar diagram shows %age apoptotic death from a representative result (2A), expressed as mean +/− SEM of at least three experiments. GSH was measured by enzymatic recycling method (2B–2C) using glutathione reductase and NADPH as described before [13]. Results are expressed as mean +/− SEM of at least three experiments. Asterisks indicate significant difference (*p≤0.05; ** p≤0.01 from control (C), ≠p≤0.05 compared to LPS-treated cells and δ p≤0.05 compared to LPS and ASA treated cells).

Figure 2A shows a significant (16%) increase in apoptotic cell death in macrophages after treatment with LPS alone. A combination of LPS and ASA further increased apoptosis in macrophages. NAC pre-treatment, on the other hand, resulted in a marked reduction in ROS production in LPS and LPS/ASA-treated cells almost to control levels.

### Effects of NAC on GSH metabolism in LPS and ASA treated macrophages

Figure 2B shows no significant change in the total cytosolic GSH pool in macrophages after treatment with LPS alone. On the other hand, a significant decrease in cytosolic GSH pool was observed when cells were treated with a combination of LPS and ASA. Treatment with NAC prior to the treatment with LPS and ASA markedly increased the cytosolic GSH pool. Mitochondrial GSH pool (Figure 2C), on the other hand, was reduced significantly (>50%) after treatment with LPS alone or the combination of LPS and ASA. NAC pre-treatment resulted in the recovery of the mitochondrial GSH pool in LPS and ASA-treated cells but not in the cells treated with LPS alone. These results suggest that mitochondrial GSH is more sensitive to LPS and ASA treatments than cytosolic GSH and NAC pre-treatment exhibited differential protection mechanism in the mitochondria and cytosol.

**Figure 3:**
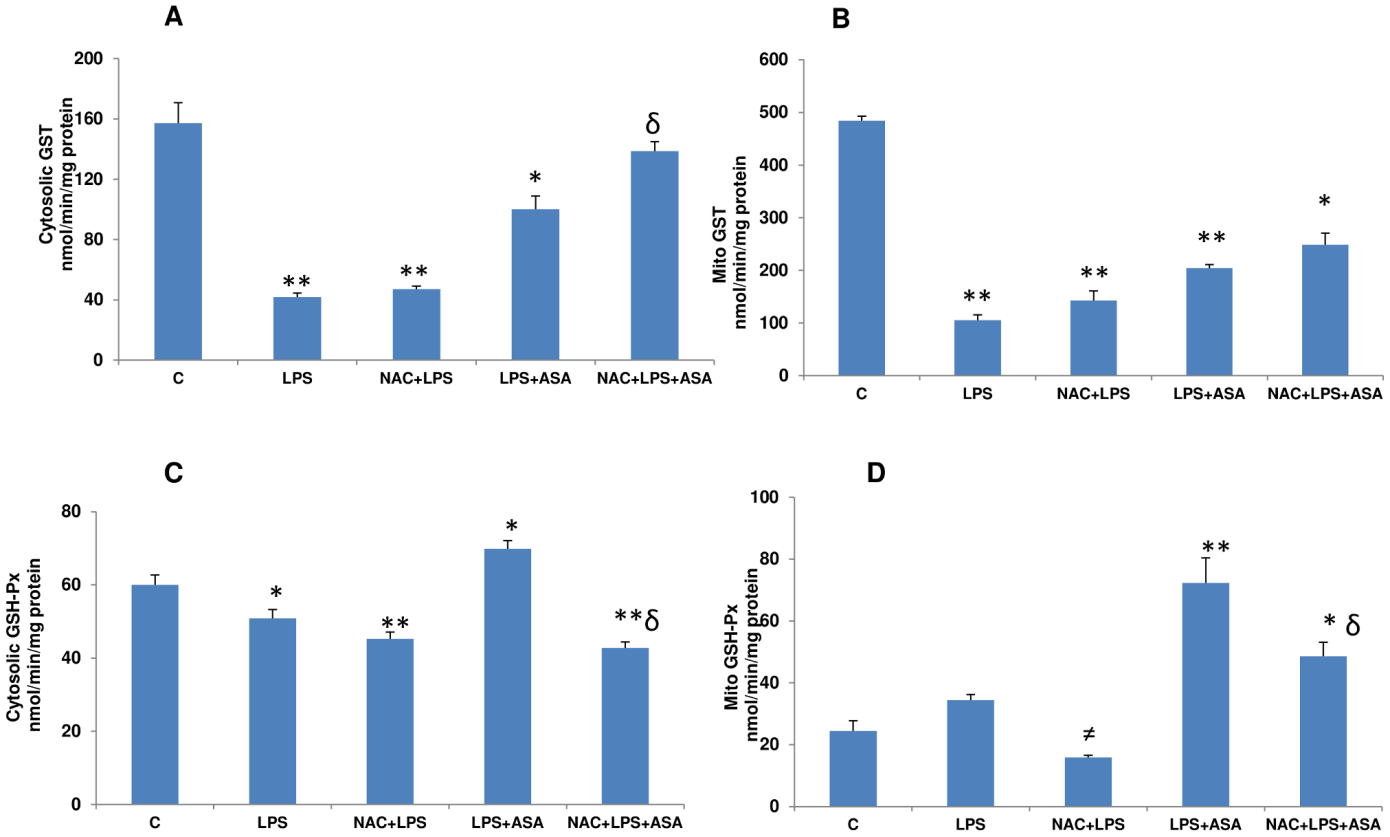
A–D: LPS- and ASA-induced alterations in GST and GSH Px activities. J774.2 macrophage cells were cultured to 80% confluence and treated with LPS alone or in combination with ASA with or without NAC as described in Materials and Methods. GST-dependent GSH conjugation (3A–3B) and GSH-Px (3C–3D) activities were measured as described before [13]. Results are expressed as mean +/− SEM of at least three experiments. Asterisks indicate significant difference (*p≤0.05, **p≤0.001 from control (C) and δ p≤0.05 compared to LPS and ASA treated cells.

The cytosolic GST conjugating as well as mitochondrial GST activities were drastically inhibited in LPS-treated macrophages (Figure 3A and B). Treatment with NAC prior to LPS treatment had no significant effect on the inhibition of GSTs in these cellular compartments. The combination of LPS and ASA also resulted in significant inhibition of GST activities. NAC pre-treatment, however, resulted in a partial recovery of GST activity which was still significantly lower in the mitochondrial fraction, compared to the cytosolic GST, which showed better recovery.

GSH-Px activity in the cytosol (Figure 3C) of macrophages was significantly elevated with LPS alone, which was further increased in combination with ASA. Pre-treatment with NAC resulted in a marginal recovery in cells treated with LPS alone whereas a significant recovery was observed with LPS and ASA. Similarly, mitochondrial GSH-Px enzyme activity (Figure 3D), was elevated with LPS alone, which was again significantly elevated with a combined treatment of LPS and ASA. A significant recovery in activity was observed with NAC pre-treatment.

### Effects of NAC on CYP450 activities in LPS and ASA-treated cells

**Figure 4:**
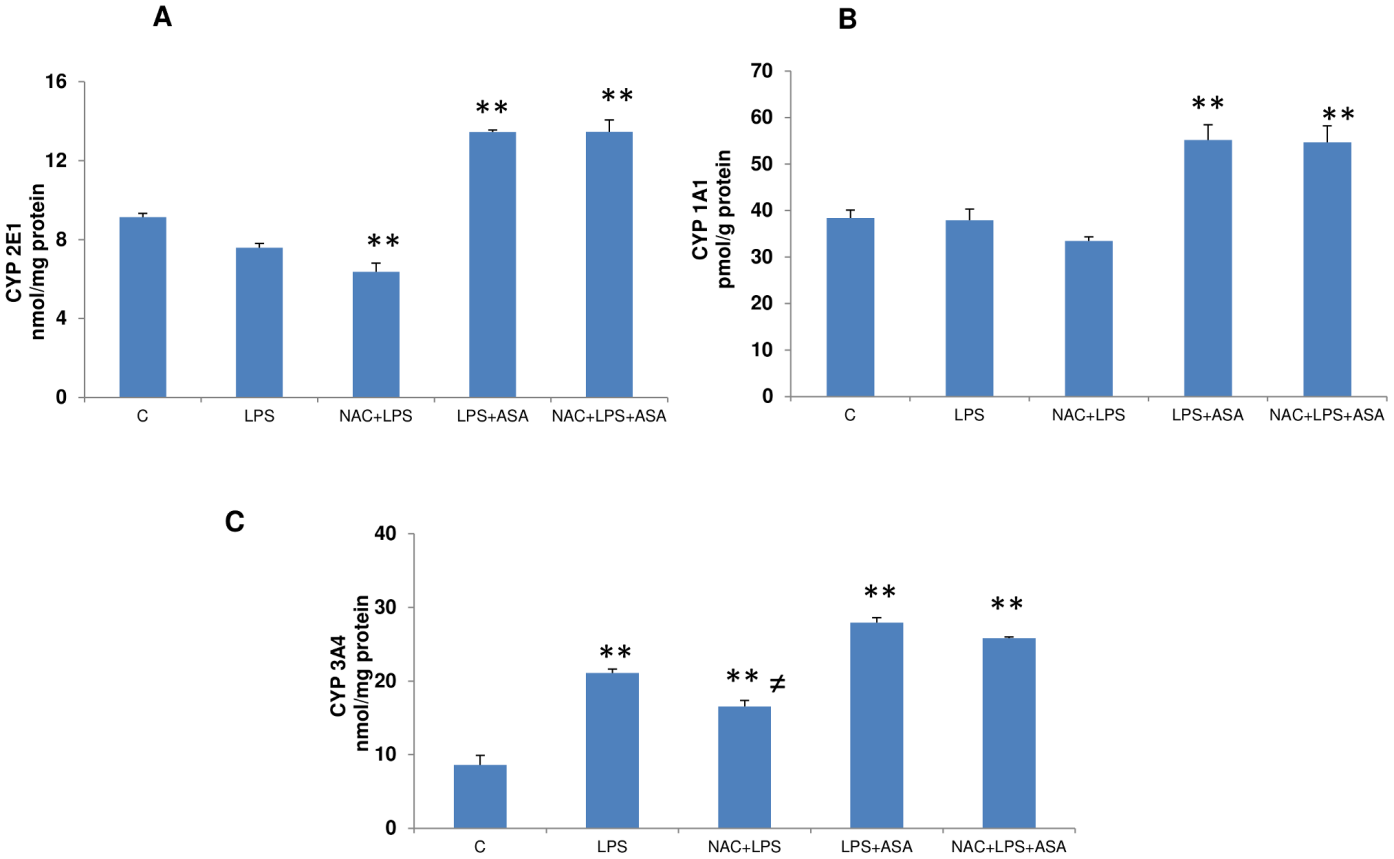
LPS- and ASA-induced alterations in CYP activities. J774.2 macrophage cells were cultured to 80% confluence and treated with LPS alone or in combination with ASA with or without NAC as described in Materials and Methods. CYP2E1 (4A), CYP 1A1 (4B) and CYP 3A4 (4C) activities were measured as described in Materials and Methods. Results are expressed as mean +/− SEM of at least three experiments. Asterisks indicate significant difference (*p≤0.05, **p≤0.001) from control (C).

CYP 2E1 activity (Figure 4A) in macrophages treated with LPS alone or pretreated with NAC was not altered significantly. On the other hand, cells treated with a combination of LPS and ASA showed a significant increase (>50%) in enzyme activity in the macrophages, which remained unaltered even after prior treatment with NAC.

Similarly, CYP 1A1 activity also increased (40%) with a combined treatment of LPS and ASA but not with LPS alone (Figure 4B). NAC pre- treatment did not affect the enzyme activity in these cells.

CYP 3A4 activity, on the other hand, was increased 2-fold when cells were treated with LPS alone with a further increase (3-fold) observed with LPS and ASA (Figure 4C). NAC pre-treatment had no significant effect on the increased enzyme activity.

### Effects of NAC on LPS-and ASA-induced cytokine production

**Figure 5:**
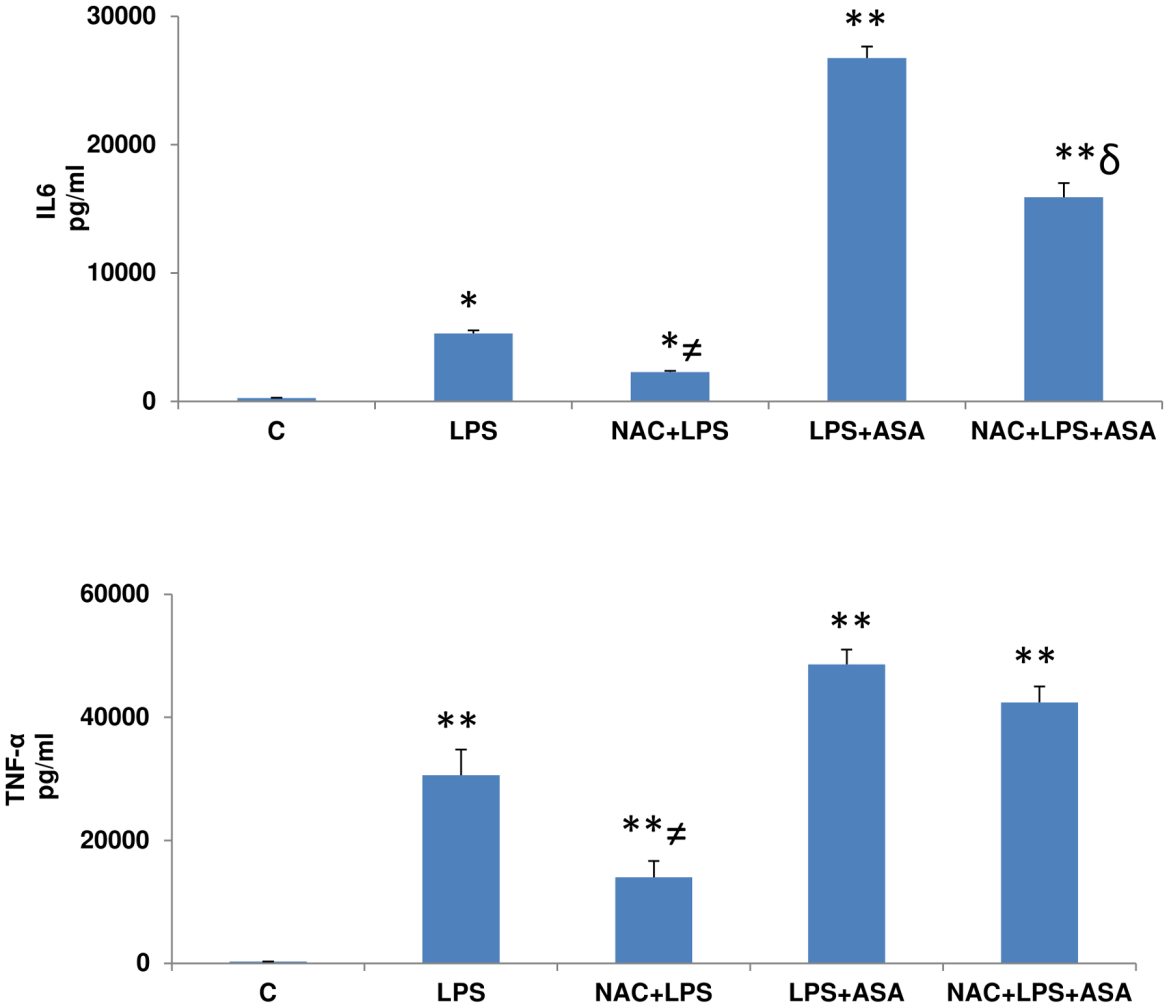
LPS- and ASA-induced alterations in cytokines. J774.2 macrophage cells were cultured to 80% confluence and treated with LPS alone or in combination with ASA with or without NAC as described above and cytokines IL6 and TNF-α were measured using standard ELISA kits as described in Materials and Methods. Results are expressed as mean +/-SEM of at least three experiments. Asterisks indicate significant difference (*p≤0.05, **p≤0.001) from control (C) and δ p≤0.05 compared to LPS and ASA treated cells.

Figure 5 shows that the levels of cytokines, IL6 and TNF-α were significantly increased with LPS treatment which further increased several fold after the combined treatment of LPS and ASA. NAC pre-treatment significantly reduced the level of the cytokines in macrophages though the levels remained significantly higher than that in the control cells.

### Effects of NAC on mitochondrial functions in LPS and ASA-treated cells

**Figure 6:**
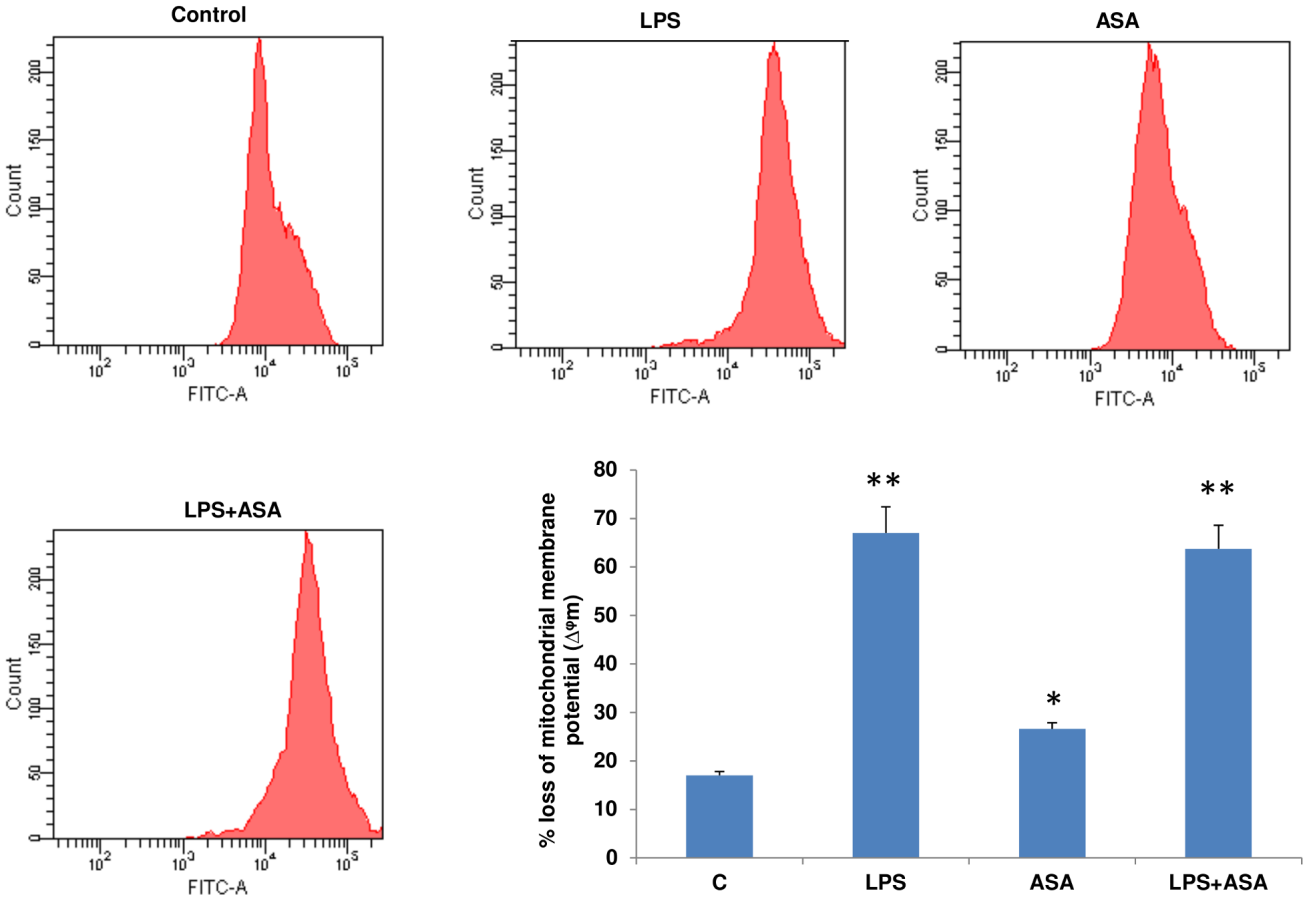
LPS- and ASA-induced alteration in mitochondrial membrane potential. J774.2 macrophage cells were cultured to 80% confluence and treated with LPS and ASA alone or in combination as described above. Mitochondrial membrane potential was measured using a cationic fluorescent dye as described before [13]. A typical histogram representative of at least three experiments showing % loss of mitochondrial membrane potential is shown. Results are expressed as mean +/−SEM of at least three experiments. Asterisks indicate significant difference (*p≤0.05, **p≤0.001) from control (C).

**Figure 7:**
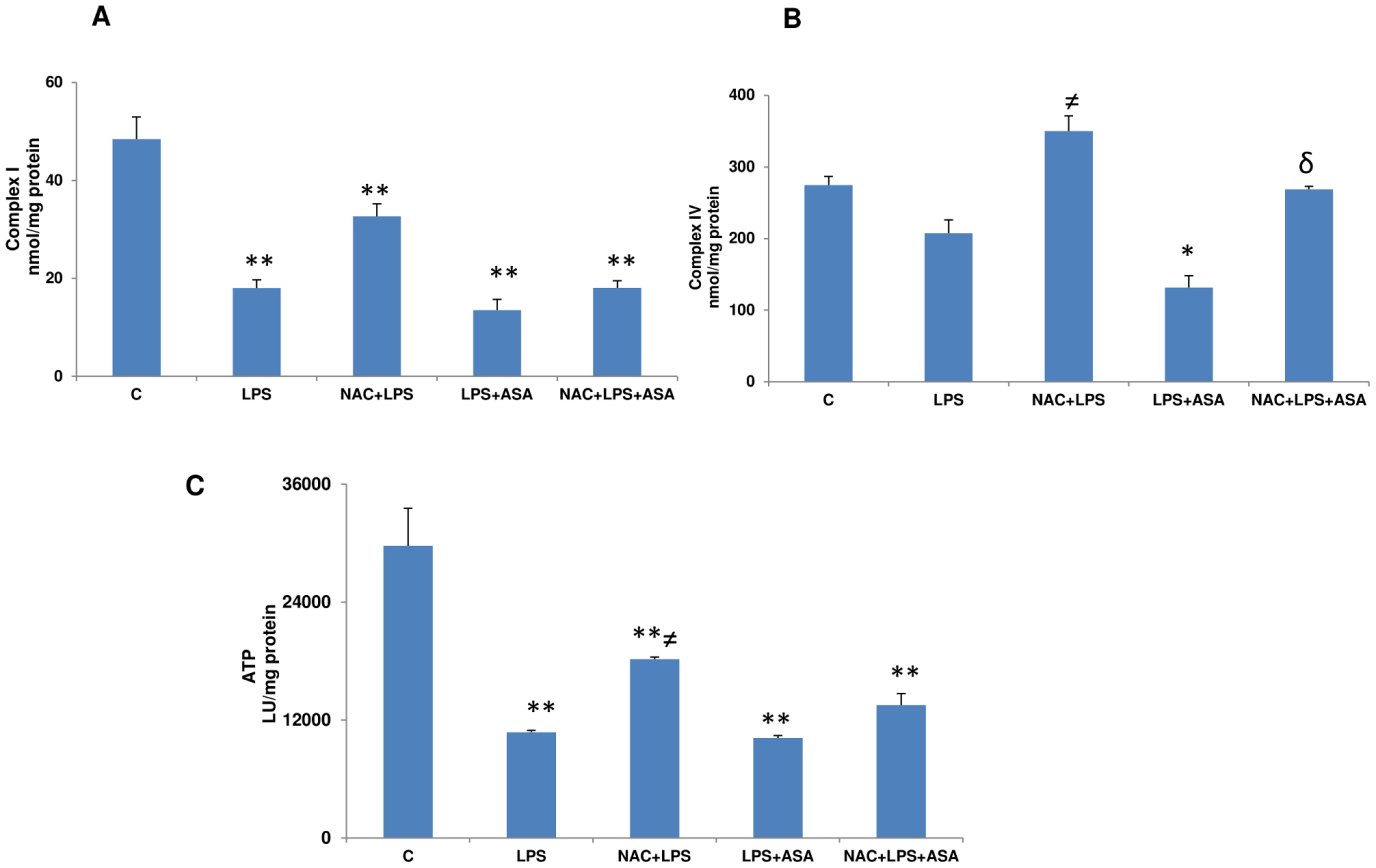
A–C: LPS- and ASA-induced alterations in mitochondrial respiratory functions. J774.2 macrophage cells were cultured to 80% confluence and treated with LPS alone or in combination with ASA with or without NAC as described above. Freshly isolated mitochondria from control and treated cells were used to assay Complex I and Complex IV activities (7A–7B) and ATP content (7C) as described before [13]. Results are expressed as mean +/−SEM of at least three experiments. Asterisks indicate significant difference (*p≤0.05, **p≤0.001) from control (C), ≠p≤0.05 compared to LPS-treated cells and δ p≤0.05 compared to LPS and ASA treated cells.

Our studies showed a significant loss in mitochondrial membrane potential (3–4 fold) after LPS treatment or after ASA treatment (about 2-fold) (Figure 6). A similar loss was observed when a combination of LPS and ASA was used. This was the basis to further study the effects of NAC on mitochondrial functions. As shown in figure 7A, Complex I activity was markedly inhibited in cells treated with LPS alone or combination with ASA. NAC pre-treatment only had a marginal effect on the recovery of this enzyme activity. Cytochrome c oxidase (Complex IV) activity (Figure 7B), on the other hand, was moderately inhibited by LPS alone but significantly by a combination of LPS and ASA treatment. NAC pre-treatment exhibited almost a complete recovery of enzyme activity in these cells.

A significant loss of ATP production in macrophages was observed after treatment with LPS alone or combination of LPS and ASA (Figure 7C) while NAC pre-treatment caused only a partial recovery.

### Effects of LPS and ASA on the expression of redox markers and apoptotic proteins

**Figure 8:**
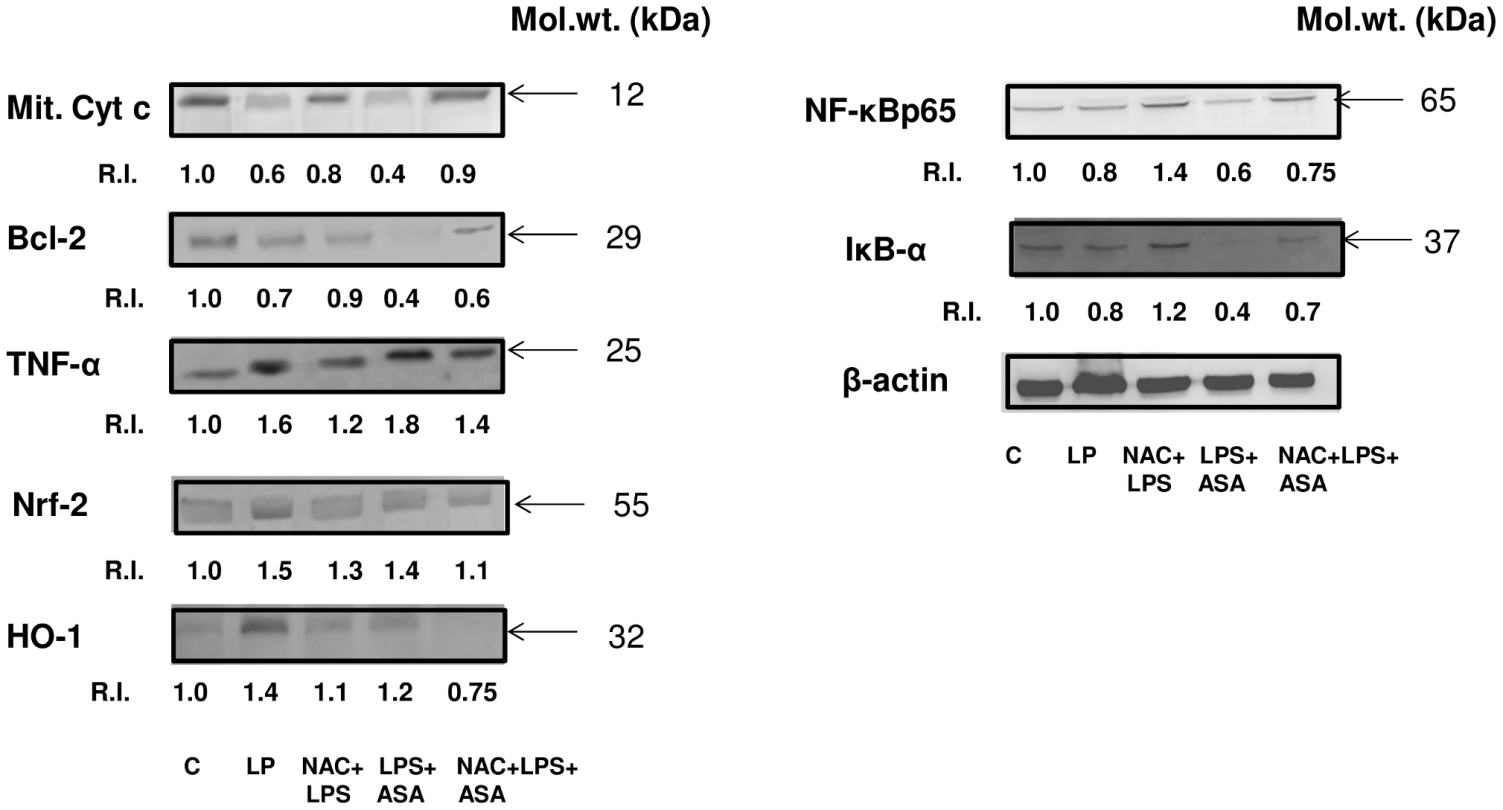
LPS- and ASA-induced alterations in protein expression. J774.2 macrophage cells were cultured to 80% confluence and treated with LPS alone or in combination with ASA with or without NAC as described above. Proteins (50–100 µg) from cell lysates were separated by SDS-PAGE analysis and transferred on to membranes (Western blot). Transferred proteins were incubated with primary antibodies against cytochrome c, Bcl-2, TNF-α, Nrf-2, HO-1, IκB-α and NF-κBp65 and antibody reacting proteins were visualized as described before [13]. Beta-actin was used as loading control. Results from representative SDS-PAGE/Western blot analysis are shown. The quantitation of protein bands is expressed as relative intensity (R.I) of the protein considering the expression of proteins in control untreated cells as 1.0. Molecular weight markers (kDa) are indicated by arrows.

As shown in Figure 8, a 40% decrease in the level of mitochondrial cytochrome c content was observed after treatment with LPS alone and a 60% decrease after the LPS and ASA treatment. A 30% and a 60% reduction in the expression of anti-apoptotic protein, Bcl-2, was also observed in the LPS alone treatment and the LPS and ASA treatment respectively. Consistent with the release of inflammatory cytokines, the expression of TNF-α was significantly higher in macrophages treated with either LPS alone or in combination with ASA. Increased expression of redox-marker proteins, Nrf-2 and HO-1, was also observed when macrophages were treated with LPS alone or in combination with ASA. NAC pre-treatment resulted in a partial or complete recovery of these apoptotic and oxidative stress marker proteins. A reduced expression of cytosolic IκB-α and NF-κBp65 was also observed when macrophages were treated with LPS alone or in combination with ASA. This suggests an increased nuclear translocation of NF-κBp65. NAC pre-reatment caused a complete recovery of NF-κB in the cytosol. These results suggest the involvement of NF-κB signaling in LPS and ASA-induced alterations in redox homeostasis in the macrophages.

## Discussion

Pathogen- and drug-induced toxicity and tissue injury are the major challenges in clinical and experimental pharmacology. Macrophages are important immune-responsive cells and have multiple roles in increasing as well as reducing the pathophysiological responses in the host [21]. The endotoxin, LPS, is released upon infection or from the gastrointestinal fauna and enters the blood stream and other tissues such as liver which also regulates the inflammatory and toxic responses towards LPS exposure [7], [22]. Inflammation-, cytokine- and endotoxin-associated idiosyncratic drug toxicities have also been reported to exhibit synergetic actions for the production of pro-inflammatory cytokines [23]–[25].We, therefore, have investigated the oxidative stress and associated metabolic complications of LPS and ASA on macrophages. Our previous study on acetaminophen showed increased mitochondrial and oxidative stress in J774.2 cells [12]. Also, using HepG2 cells, we have provided evidence that ASA induces cell cycle arrest and mitochondrial dysfunction accompanied by apoptosis [11], [13]. There is extensive evidence that inflammatory, pharmacological and toxicological properties of nonsteroidal anti-inflammatory drugs, (NSAIDs), are regulated by macrophage-produced cytokines and liver cells and that there are cooperative roles of inflammation and oxidative stress in the pathogenesis of disease [3], [23]-[24]. Acetaminophen has also been shown to act synergistically with LPS for the production of pro-inflammatory cytokines in murine macrophages [24]. Similarly, ASA has also been shown to increase synthesis of interleukins and TNF-α [25], [26]. It has also been reported that high doses of ASA are needed to inhibit pro-inflammatory cytokine production while low-to-moderate doses enhances their production [27].

We have observed an increased ROS production by LPS in macrophages and have shown that ASA has synergistic effects when treated in conjunction with LPS. Apparently, both mitochondrial and extra-mitochondrial ROS production were cooperatively increased after treatment with LPS. The present study has also shown a compromised GSH-dependent redox metabolism, particularly in the mitochondria, as observed by a marked reduction in LPS/ASA-induced GSH concentration. Pre-treatment with NAC, an antioxidant and a scavenger of ROS, on the other hand, caused some partial recovery from the drug- and LPS-induced alterations in the GSH level. NAC pre-treatment also showed inhibition in the increased production of ROS. However, apoptotic cell death could not be recovered by NAC pre-treatment. Both mitochondrial and extra-mitochondrial GSH-conjugating activity was markedly inhibited after ASA and LPS treatment. Only partial recovery by NAC pre-treatment was observed. GSH-Px activity in the cytosolic and the mitochondrial fraction was elevated by LPS and ASA treatment while pre-treatment with NAC resulted in partial recovery of the activity. CYP 2E1 is the most important cytochrome P450 in the regulation of cellular ROS production [5]. In our study on macrophages, the CYP 2E1 activity was only increased after the combined treatment of LPS and ASA but not with LPS alone. NAC pre-treatment did not alter the increased CYP 2E1 activity in LPS and ASA treated cells. Similarly, CYP 1A1 activity was also increased when macrophages were treated with LPS and ASA but not with LPS alone. CYP 3A4 activity, on the other hand, was markedly increased on treatment with LPS alone or in combination with ASA. NAC pre-treatment had little effect on the increased CYP 3A4 activity. These results suggest differential roles of the different P450s in potentiating the endotoxin and ASA-induced oxidative stress and toxicity. It has been reported that alterations in GSH metabolism, cytokines and TNF-α production, and CYP activities by LPS and ASA may have implications in altering the cell signaling, oxidative stress, mitochondrial functions and apoptosis [11]–[13], [28]–[29].

We had earlier reported inhibition of aconitase activity by ASA treatment in HepG2 [13]. Our preliminary results using macrophages and HepG2 cells have shown that LPS and ASA treatment alone or in combination resulted in increased ROS/NO production, lipid peroxidation and inhibition of ROS-sensitive mitochondrial aconitase [30] suggesting mitochondrial dysfunction. In the present study, we have further confirmed that treatment with LPS alone or in combination with ASA induced a loss of mitochondrial membrane potential accompanied by inhibition in respiratory Complex I activity and ATP synthesis. NAC pre-treatment caused only a partial recovery of ATP synthesis whereas a significant recovery of Complex I activity was observed when cells were pretreated with NAC. It has been shown that the elevation of mitochondrial GSH-Px activity and ROS production enhances the glutathionylation of the respiratory complexes and thus alters mitochondrial coupling which in turn contributes to decreased ATP synthesis [31]. Increased expression of oxidative stress marker proteins such as HO-1 and redox-sensitive transcription factors Nrf-2 and NF-κB by treatment with LPS alone or in combination with ASA were also confirmed by Western blot analysis. Similarly, a decrease in the anti-apoptotic protein, Bcl-2, and mitochondrial cytochrome c has suggested an increase in apoptotic cell death after treatment with LPS alone or in combination with ASA. NAC pre-treatment resulted in partial recovery in the expression of these redox-sensitive proteins. Mitochondrial dysfunction and increased oxidative stress by treatment with LPS alone or in conjunction with ASA in macrophages have supported the evidence that increased oxidative stress triggers the apoptotic events in macrophages [11]–[13], [28]–[30], [32]–[33].

In summary, ASA treatment appears to have facilitated and enhanced the responses in macrophages towards endotoxins, thus providing a better approach for therapeutic intervention against pathogen-induced toxicity. NAC pretreatment had some protective effects against LPS and ASA-induced toxicity.
